# Low-dose Diosbulbin-B (DB) activates tumor-intrinsic PD-L1/NLRP3 signaling pathway mediated pyroptotic cell death to increase cisplatin-sensitivity in gastric cancer (GC)

**DOI:** 10.1186/s13578-021-00548-x

**Published:** 2021-02-12

**Authors:** Chunfeng Li, Junqiang Qiu, Yingwei Xue

**Affiliations:** 1 Gastrointestinal Surgical Ward, Harbin Medical University Cancer Hospital, Haping Road 150, Harbin, 150081 Heilongjiang China; 2 Department of Inorganic Chemistry and Analytical Chemistry, School of Pharmacy, Hainan Medical University, Xueyuan Road No. 3, Haikou, 571199 Hainan China

**Keywords:** Gastric cancer, Cancer stem cells, Diosbulbin-B, Pyroptosis, Apoptosis

## Abstract

**Background:**

Emerging evidences suggests that Diosbulbin-B (DB) is effective to improve cisplatin (DDP)-sensitivity in gastric cancer (GC), but its molecular mechanisms were not fully delineated, and this study managed to investigate this issue.

**Methods:**

Genes expressions were determined by Real-Time qPCR and Western Blot at transcriptional and translational levels. Cell proliferation and viability were evaluated by cell counting kit-8 (CCK-8) and trypan blue staining assay. Annexin V-FITC/PI double staining assay was used to examine cell apoptosis. The Spheroid formation assay was used to evaluated cell stemness. The xenograft tumor-bearing mice models were established, and the tumors were monitored and the immunohistochemistry (IHC) was employed to examine the expressions and localization of Ki67 protein in mice tumor tissues.

**Results:**

Low-dose DB (12.5 μM) downregulated PD-L1 to activate NLRP3-mediated pyroptosis, and inhibited cancer stem cells (CSCs) properties, to sensitize cisplatin-resistant GC (CR-GC) cells to cisplatin. Mechanistically, the CR-GC cells were obtained, and either low-dose DB or cisplatin alone had little effects on cell viability in CR-GC cells, while low-dose DB significantly induced apoptotic cell death in cisplatin treated CR-GC cells. In addition, low-dose DB triggered cell pyroptosis in CR-GC cells co-treated with cisplatin, which were abrogated by silencing NLRP3. Next, CSCs tended to be enriched in CR-GC cells, instead of their parental cisplatin-sensitive GC (CS-GC) cells, and low-dose DB inhibited spheroid formation and stemness biomarkers (SOX2, OCT4 and Nanog) expressions to eliminate CSCs in CR-GC cells, which were reversed by upregulating programmed death ligand-1 (PD-L1). Furthermore, we proved that PD-L1 negatively regulated NLRP3 in CR-GC cells, and low-dose DB activated NLRP3-mediated pyroptotic cell death in cisplatin treated CR-GC cells by downregulating PD-L1. Also, low-dose DB aggravated the inhibiting effects of cisplatin on tumorigenesis of CR-GC cells in vivo.

**Conclusions:**

Collectively, low-dose DB regulated intrinsic PD-L1/NLRP3 pathway to improve cisplatin-sensitivity in CR-GC cells, and this study provided alternative therapy treatments for GC.
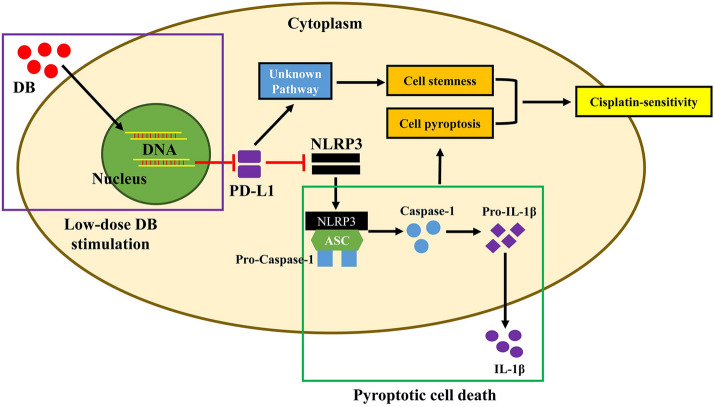

## Introduction

The therapeutic efficacy of the current chemical drugs for gastric cancer (GC) were seriously limited as the results of chemo-resistance [1–3], hence, the development of new anti-tumor drugs for GC treatment became urgent and necessary. Based on the information provided by the previous publications [4, 5], one of the Chinese medicine, *Dioscorea bulbifera *L., had been widely used for cancer treatment in Asia, and researchers had extracted the main anti-tumor compound, Diosbulbin-B (DB), from this Chinese herb to treat cancers [5–7]. However, high-dose DB-induced hepatotoxicity [8], and the ineffectiveness of low-dose DB for cancer treatment seriously limited its utilization for GC treatment in clinic [5]. To solve this issue, our preliminary work tried to use low-dose DB combined with genes manipulation to treat GC, and the results indicated that this strategy successfully hampered GC development, but had little detrimental effects on human normal hepatocytes (L-02) [5]. The above information enlightened us to investigate the possibility whether low-dose DB treatment was capable of improving the chemo-sensitivity of GC cells to the current chemical drugs. To achieve this, this study selected cisplatin (DDP), one of the most common chemotherapeutic drug for GC treatment [9, 10], for further investigations.

Cancer stem cells (CSCs) were a subgroup of cancer cells characterized by high self-renewal abilities [11, 12], which sustained the heterogeneity of tumor cells and contributed to metastasis and bad prognosis in GC patients [13, 14]. In addition, recent data indicated that CSCs were pivotal for sustaining cisplatin-resistance in multiple cancers, such as head and neck squamous cell carcinoma (HNSCC) [15], lung cancer [16] and GC [17, 18]. Specifically, Peng C et al. found that restriction of CSCs features enhanced cisplatin sensitivity [18], and Zhang L et al. validated that induction of CSCs properties increased cisplatin-resistance in GC cells [17], which suggested that elimination of CSCs was an ideal strategy to improve cisplatin-sensitivity in GC [19]. By conducting the preliminary experiments, we surprisingly found that low-dose DB regulated CSCs properties in GC cells, however, up until now, no existed literatures reported the link between DB and cell stemness. Programmed death ligand-1 (PD-L1) functioned as an oncogene to facilitate GC progression [20, 21], which was also associated with immune evasion in tumor microenvironment [22, 23]. Aside from that, Y Zuo et al. validated that PD-L1 promoted cisplatin-resistance in ovarian cancer [24], and Fang Wei et al. found that upregulation of PD-L1 specifically promoted CSCs expansion in colorectal cancer [25]. Interestingly, a translational analysis from the ITACA-S trial (https://clinicaltrials.gov/, Identifier: NCT01640782) evidenced that PD-L1 was a potential predictor of benefit from intensive cisplatin-chemotherapy [26]. Notably, our preliminary experiments showed that low-dose DB regulated PD-L1 expressions in GC cells.

Cell pyroptosis was a type of cell death featured by NLRP3 inflammasome activation and pro-inflammatory cytokines secretion [27, 28], and induction of pyroptotic cell death was proved to be an effective strategy to eliminate cancer cells [29, 30]. For example, myeloid phosphate and tension homology deleted on chromosome ten (PTEN) promoted chemotherapy-induced NLRP3 mediated cell pyroptosis to inhibit cancer development [29], and activation of NLRP3-mediated pyroptotic cell death enhanced the cytotoxic effects of cisplatin in non-small cell lung cancer (NSCLC) [30]. Of note, Bala et al. validated that there existed an intrinsic PD-L1/NLRP3 signaling pathway in melanoma cells [31], and the correlations between PD-L1 and NLRP3 had also been observed in interferon-γ (IFN-γ) induced myotubes [32]. However, the detailed information for the PD-L1-NLRP3 pathway had not been deeply investigated in other types of cells, such as GC cells. Based on this, we validated that PD-L1 negatively regulated NLRP3 in GC cells in our preliminary work, which rendered the possibility that targeting PD-L1-NLRP3 pathway was novel to enhance cisplatin-sensitivity in GC cells. In addition, DB exerted anti-tumor effects by regulating multiple tumor-associated genes [5, 7], and our preliminary data suggested that low-dose DB triggered cell pyroptosis in cisplatin treated GC cells, and potentially regulated PD-L1-NLRP3 pathway.

Taken together, this study managed to investigate the potential utilization of low-dose DB combined with traditional chemical drugs to treat GC in vitro and in vivo, and uncovered the potential underlying mechanisms. This study will broaden our knowledge in this field, and provide potential therapeutic agents for GC treatment in clinic.

## Materials and methods

### Cell culture and treatment

The cisplatin-resistant GC (CR-GC) cell lines (SGC7901/CDDP, BGC823/CDDP) and their parental cisplatin-sensitive GC (CS-GC) cell lines (SGC7901 and BGC823) were obtained from the Resistant Cancer Cell Line Collection (http://www.kent.ac.uk/) and American Type Culture Collections (ATCC, USA), respectively. All the cells were cultured under standard culture conditions with 37 ℃ humidified air containing 5% CO_2_ and cultivated in the RPMI-1640 medium (Gibco, USA) containing 10% fetal bovine serum (FBS, Gibco, USA). After that, the CR-GC cells were subjected to cisplatin (20 μg/ml) combined with low-dose DB (12.5 μM) for 0 h, 24 h, 48 h and 72 h, respectively. To induce the acquired cisplatin-resistant GC (ACR-GC) cells, the parental CS-GC cells were exposed to continuous low-dose cisplatin (from 0.5 to 5 μg/ml, for 80 days) in a step-wise manner based on the experimental procedures provided by the previous publications [9, 10]. In addition, the PD-L1 recombinant protein were bought from BioVision (#P1023-10, USA).

### Vectors transfection

The small interfering RNAs (siRNA) for PD-L1 and NLRP3 were designed and synthesized by Sangon Biotech (Shanghai, China), and the PD-L1 overexpression vectors were constructed by Vazamy (Shanghai, China). After that, the commercial Lipofectamine 2000 reagent (Invitrogen, CA, USA) was purchased to deliver the above vectors into CR-GC cells, the detailed information for vectors transfection procedures were documented in the producer’s instructions. The sequence information for the above plasmids had been included in Additional file 1: Table S1.

### Cell counting kit-8 (CCK-8) assay

The CR-GC cells were pre-transfected with differential vectors for genes manipulation, and were administered with cisplatin and DB stimulation, respectively. After that, the cells were cultured in the incubators for 0 h, 24 h, 48 h and 72 h, respectively, and the commercial CCK-8 assay kit (Beyotime, Shanghai, China) was obtained to measure cell proliferation abilities based on the manufacturer’s protocol.

### Trypan blue staining assay

The CR-GC cells were exposed to differential treatments, harvested, prepared and stained with trypan blue reaction buffer (Sigma-Aldrich, USA) at the concentration of 0.4% for 20 min at room temperature. After that, the cells were observed under a light microscope, and the dead blue cells were counted. Finally, the cell viability was calculated by using the following formula that: cell viability (%) = (total cells − dead blue cells)/total cells × 100%.

### Annexin V-FITC/PI double staining assay

The CR-GC cells were pre-transfected with different vectors, and administered with cisplatin (20 μg/ml) combined with low-dose DB (12.5 μM) for 48 h, the commercial Apoptosis Detection kit was purchased from BD Bioscience (USA) to measure cell apoptosis ratio in keeping with the producer’s protocol. Specifically, the CR-GC cells were harvested, washed and stained with Annexin V-FITC and PI staining solution for 40 min at room temperature without light exposure. Finally, the apoptotic cell proportions were determined by using the flow cytometer (FCM) purchased from ThermoFisher Scientific (USA).

### Real-time qPCR

The CR-GC cells were prepared, and a commercial TRIzol reagent (Invitrogen, USA) was obtained to extract the total RNA from the cells in keeping with the producer’s protocol. After that, the Real-Time qPCR kit (Invitrogen, USA) was employed to to examine the mRNA levels of PD-L1, SOX2, OCT4, Nanog and β-actin in the CR-GC cells according to the experimental procedures provided by the previous publications [9, 10]. The primer sequences for Real-Time qPCR were included in Additional file 1: Table S2.

### Western blot analysis

We bought the RIPA lysis buffer (Beyotime, Shanghai, China) to extract the total protein from CR-GC cells and mice tumor tissues based on the experimental procedures provided by the manufacturer. After that, the protein levels of Cyclin D1, CDK2, β-actin, PD-L1, SOX2, OCT4, Nanog, NLRP3, ASC, IL-1β, IL-18, cleaved Caspase-3 (p17) and Bax were determined by using the Western Blot analysis, and the experimental procedures had been well documented in the previous publications [9, 10]. Besides, the detailed information for the involved primary antibodies were documented in Additional file 1: Table S3.

### Spheroid formation assay

The ACR-GC cells, CR-GC cells and CS-GC cells were cultured under the standard conditions with RPMI-1640 medium (Gibco, USA), and the spheroid formation abilities of the above cells were measured by using the spheroid formation assay, and the detailed experimental procedures had been well recorded in the previous publication [33–35]. Briefly, the cells were washed, centrifuged and grown in the serum-free RPMI-1640 medium containing 10 mM 2-(4-(2-Hydroxyethyl)-1-piperazinyl) ethanesulfonic acid (HEPES), B27 (0.02%), epidermal growth factor (EGF, 20 ng/mL) and basic fibroblast growth factor (bFGF, 10 ng/mL) for 14 days cultivation. After that, the cell spheres were observed and counted under the light microscope to evaluate cell stemness.

### Xenograft tumor-bearing mice models

The BALB/c mice (aged 4–6 weeks) were purchased from the Research Animal Center of Harbin Medical University, and all the animals were fed and housed with standard conditions. Next, the cells were pre-transfected differential vectors, including PD-L1 overexpression and downregulation vectors, and the cells were implanted into the dorsal flank regions of the mice at the density of 2 × 10^7^ cells per mice. The mice tumor was monitored, and tumor volume was measured every 5 days. At 30 days post-injections, the mice were sacrificed and the tumors were obtained, photographed and weighed to reflect tumorigenesis of CR-GC cells in vivo. All the animal experiments were approved by the Ethics Committee for Animal Experimentation of Harbin Medical University.

### In vivo* extreme limiting dilution analysis (ELDA) for cell stemness*

The CR-GC cells with or without PD-L1 overexpression vectors were injected into the dorsal flank regions of the mice at the number of 1 × 10^4^ cells, 1 × 10^5^ cells and 1 × 10^6^ cells, respectively. The mice were divided into 3 groups, including control, DB and DB + OE-PD-L1 group. The formation of the tumors were monitored and documented regularly at day 8, 11, 14, 17, 20, 23, 26, 29, 32 and 35 post-injection to evaluate cell stemness in vivo. All the animal experiments were approved by the Ethics Committee for Animal Experimentation of Harbin Medical University.

### Immunohistochemistry (IHC)

The mice tumor tissues were collected, fixed with formaldehyde, embedded by paraffin, and spliced into sections with 5 μm thickness. After that, according to the experimental procedures provided by the previous work [33], the IHC was conducted to examine the expressions and localization of Ki67 protein in mice tumor tissues, which could reflect the proliferation abilities of CR-GC cells in vivo.

### Statistical analysis

The data involved in this study was presented as Means ± Standard Deviation (SD), and analyzed by using the commercial SPSS 18.0 statistical software. Specifically, the means in two group was compared by using Student’s t-test, and one-way ANOVA analysis was used to compare the means from multiple groups (> 2). One individual experiment was repeated at least 3 times in our work, and *P* < 0.05 was regarded as statistical significance and marked with “*”.

## Results

### Low-dose DB sensitized CR-GC cells to cisplatin treatment

**Fig. 1:**
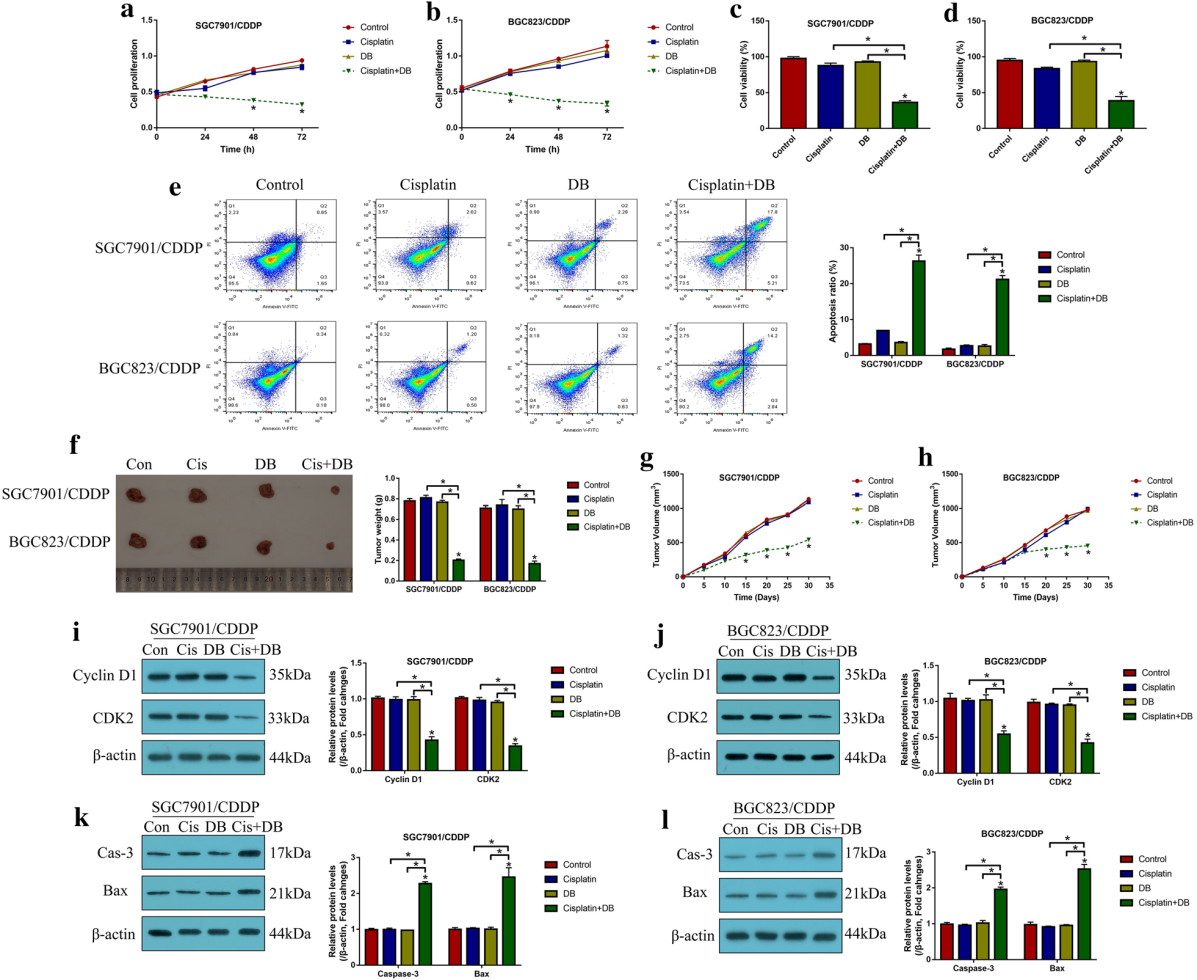
Low-dose DB sensitized CR-GC cells to cisplatin stimulation. The CR-GC cell lines (SGC7901/CDDP and BGC823/CDDP) were subjected to low-dose DB and high-dose cisplatin stimulation for 0 h, 24 h, 48 h and 72 h, respectively. **a**, **b** Cell proliferation and **c**, **d** viability were examined by CCK-8 assay and trypan blue staining assay. **e** Cell apoptosis was examined by Annexin V-FITC/PI double staining method. The xenograft tumor bearing mice models were established, and **f** tumor weight and **g**, **h** volume were examined, respectively. Western Blot analysis was conducted to determine the expression levels of **i**, **j** Cyclin D1 and CDK2, and **k**, **l**) cleaved Caspase-3 and Bax in CR-GC cells. (Note: “Con” indicated “Control”, “Cis” suggested “Cisplatin”, “DB” represented “Low-dose DB”, “Cis + DB” represented “Cisplatin plus low-dose DB stimulation”). Each experiment repeated at least three times. *P < 0.05

DB had been used for cancer treatment [36, 37], and the previous data from our team indicated that low-dose DB (12.5 μM) was advantageous for GC treatment to avoid DB-induced hepatotoxicity [36], which rendered the possibility that low-dose DB (12.5 μM) might be a novel strategy to increase sensitivity of CR-GC cells to the traditional chemotherapeutic drugs, such as cisplatin. To validate this speculation, the established CR-GC cells (SGC7901/CDDP and BGC823/CDDP) were treated with cisplatin (20 μg/mL) combined with low-dose DB (12.5 μM) for 0 h, 24 h, 48 h and 72 h, respectively, according to our preliminary experiments (data not shown). As shown in Fig. 1a–d, CR-GC cells were resistant to cisplatin treatment, and low-dose DB alone did not influence cell proliferation and viability in CR-GC cells (*P* > 0.05). Interestingly, DB combined cisplatin treatment significantly hindered CR-GC cell growth in vitro (*P* < 0.05, Fig. 1a–d). Consistently, by examining cell apoptosis, we found that low-dose DB triggered apoptotic cell death in cisplatin treated CR-GC cells (*P* < 0.05, Fig. 1e). Furthermore, the CR-GC cells (SGC7901/CDDP and BGC823/CDDP) were employed to establish xenograft tumor-bearing mice models, and we found that DB and cisplatin co-treatment significantly inhibited tumor weight (*P* < 0.05, Fig. 1f, Additional file 1: Figure S3) and volume (*P* < 0.05, Fig. 1g, h) to hamper tumorigenesis of the CR-GC cells in vivo. In addition, the mice tumor tissues were collected and prepared, and our following results indicated that the expression levels of Cyclin D1 and CDK2 (*P* < 0.05, Fig. 1i, j), and Ki67 (Additional file 1: Figure S1) were decreased, while Caspase-3 and Bax were increased (*P* < 0.05, Fig. 1k, l) by co-treating CR-GC cells with DB and cisplatin. The data in Fig. 1 and Additional file 1: Figure S1 suggested that low-dose DB triggered apoptotic cell death to enhance the cytotoxic effects of cisplatin on CR-GC cells. Moreover, the data in Additional file 1: Figure S5 showed that both cisplatin and DB treatment did not induce p-MLKL expression (Additional file 1: Figure S5), those data, together with the data in Fig. 1e, indicated that cisplatin-DB treatment had no impacts on cell necroptosis in CR-GC cells.

### Induction of apoptosis and pyroptosis by low-dose DB-cisplatin co-treatment in CR-GC cells

**Fig. 2:**
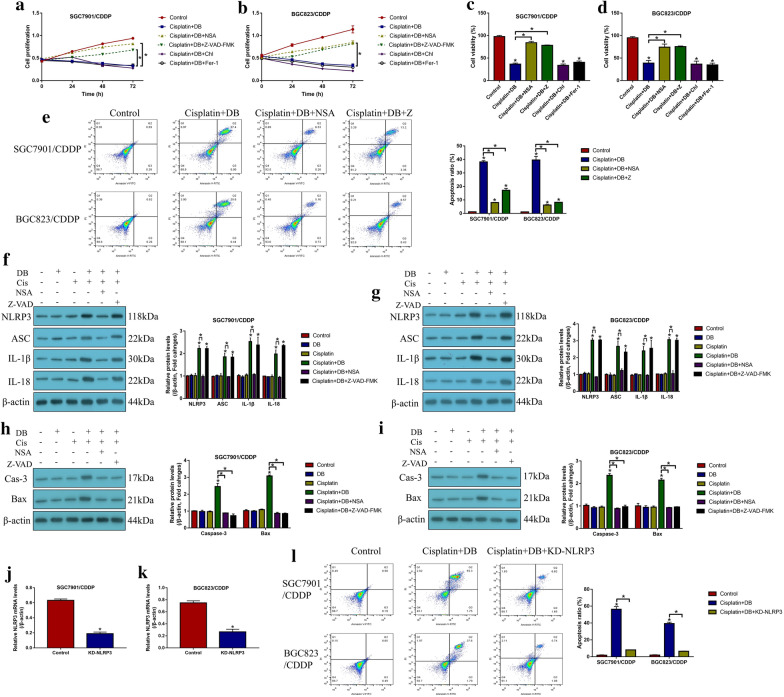
Low-dose DB increased cisplatin-sensitivity in CR-GC cells by inducing apoptotic and pyroptotic cell death. **a**, **b** cell proliferation was determined by CCK-8 assay, and **c**, **d** cell viability was measured by trypan blue staining assay. **e** Annexin V-FITC/PI double staining assay was performed to examine cell apoptosis ratio in CR-GC cells. Western Blot analysis was conducted to examine the expression levels of **f**, **g** pyroptosis associated signatures (NLRP3, ASC, IL-1β and IL-18) and **h**, **i** apoptosis associated proteins (cleaved Caspase-3 and Bax) in CR-GC cells. **j**, **k** The silencing vectors for NLRP3 were delivered into CR-GC cells. **l** Cell apoptosis was examined by Annexin V-FITC/PI double staining method. Each experiment repeated at least three times. *P < 0.05

Since cisplatin inhibited cancer progression by inducing various types of cell death, including apoptosis, pyroptosis, ferroptosis and autophagy. To investigate by which types of cell death were induced by low-dose DB in cisplatin treated CR-GC cells, the CR-GC cells were pretreated with the inhibitors for pyroptosis (Necrosulfonamide, NSA) and apoptosis (Z-VAD-FMK), autophagy (Chloroquine) and ferroptosis (Ferrostatin-1, Fer-1), respectively. The results showed that merely NSA and Z-VAD-FMK, instead of Chloroquine and Fer-1, abrogated the inhibiting effects of DB-Cisplatin combined therapy on cell proliferation (*P* < 0.05, Fig. 2a, b) and viability (*P* < 0.05, Fig. 2c, d). Also, blockage of apoptosis and pyroptosis decreased cell apoptosis ratio in DB-cisplatin co-treated CR-GC cells (*P* < 0.05, Fig. 2e), suggesting that low-dose DB aggravated the inhibiting effects of cisplatin on CR-GC cells in a apoptosis- and pyroptosis-dependent manner. Further experiments were performed to uncover the detailed mechanisms, and we found that low-dose DB combined with cisplatin increased the expression levels of pyroptosis signatures (NLRP3, ASC, IL-1β and IL-18) (*P* < 0.05, Fig. 2f, g) and apoptosis (cleaved Caspase-3 and Bax) (*P* < 0.05, Fig. 2h, i) in CR-GC cells. Interestingly, the inhibitor for pyroptosis (NSA) decreased the expression levels of both pyroptosis and apoptosis signatures (*P* < 0.05, Fig. 2f–i), while the apoptosis inhibitor (Z-VAD-FMK) had little effects on cell pyroptosis in DB-cisplatin treated CR-GC cells (*P* < 0.05, Fig. 2f–i), indicating that low-dose DB induced cell apoptosis in cisplatin treated GC cells through triggering pyroptotic cell death. Next, the siRNA for NLRP3 were transfected into CR-GC cells to knock-down NLRP3 (*P* < 0.05, Fig. 2j, k), the results showed that NLRP3 ablation abrogated the promoting effects of low-dose DB-induced cell death in cisplatin treated CR-GC cells (*P* < 0.05, Fig. 2l), implying that low-dose DB sensitized CR-GC cells to cisplatin by activating NLRP3 mediated pyroptosis.

### Enrichment of CSCs in CR-GC cells and continuous low-dose cisplatin stimulated CS-GC cells

**Fig. 3:**
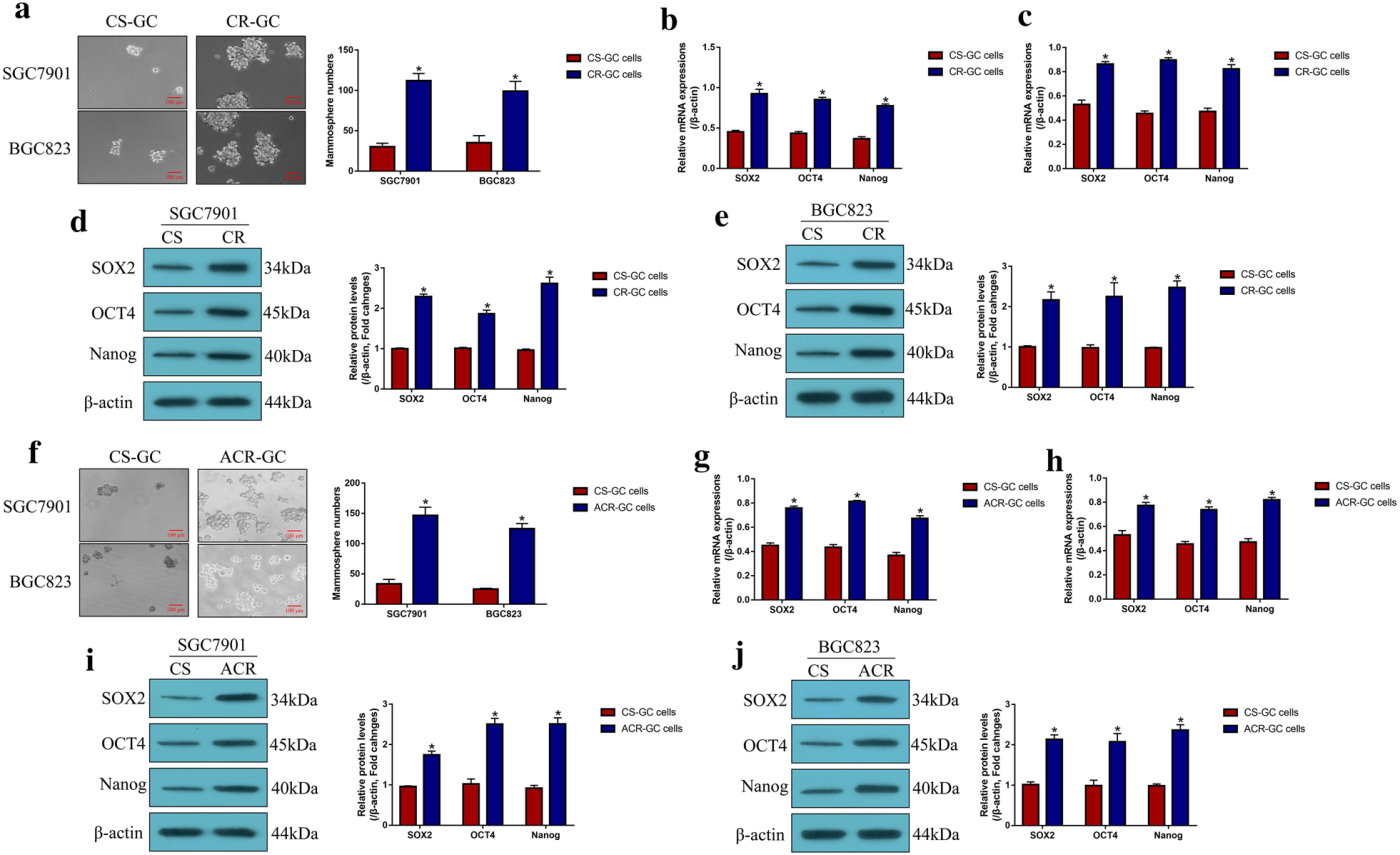
Cancer stem cells (CSCs) tended to be enriched in CR-GC cells, instead of CS-GC cells. **a** Spheroid formation abilities of the GC cells were measured by using the spheroid formation assay. The expression levels of CSCs associated signatures (SOX2, OCT4 and Nanog) were determined by using the **b**, **c** Real-Time qPCR at mRNA levels and **d**, **e** Western Blot analysis at protein levels, respectively. Also, the parental CS-GC cells were subjected to continuous low-dose cisplatin pressure to induce acquired cisplatin-resistant (ACR) GC cells, and the **f** spheroid formation abilities and **g**–**j** the expression levels of stemness signatures (SOX2, OCT4 and Nanog) were examined. Each experiment repeated at least three times. *P < 0.05

Previous data indicated that CSCs were enriched in cancer cells after long-term cisplatin stimulation, which increased resistance of cancer cells to cisplatin by sustaining tumor heterogeneity. Expectedly, this study validated that CR-GC cells (SGC7901CDDP and BGC823CDDP) were prone to form spheres compared to their corresponding parental CS-GC cells (SGC7901 and BGC823) (*P* < 0.05, Fig. 3a), and the stem cell markers (SOX2, OCT4 and Nanog) were also upregulated in CR-GC cells instead of CS-GC cells (*P* < 0.05, Fig. 3b–e), suggesting that CSCs were enriched in CR-GC cells. Also, to validate the above results, according to the previous study, the parental CS-GC cells (SGC7901 and BGC823) were exposed to continuous low-dose cisplatin treatment to simulate the generation of acquired cisplatin resistant GC (ACR-GC) cells in vitro. As expected, we observed that continuous low-dose cisplatin pressure increased the spheroid formation abilities (*P* < 0.05, Fig. 3f), and promoted SOX2, OCT4 and Nanog expressions (*P* < 0.05, Fig. 3g–j) in ACR-GC cells, compared to the CS-GC cells. In general, analysis of the above data in Fig. 3 suggested that continuous cisplatin pressure induced CSCs properties in CR-GC and ACR-GC cells.

### Low-dose DB regulated CSCs properties in CR-GC cells by inhibiting PD-L1

**Fig. 4:**
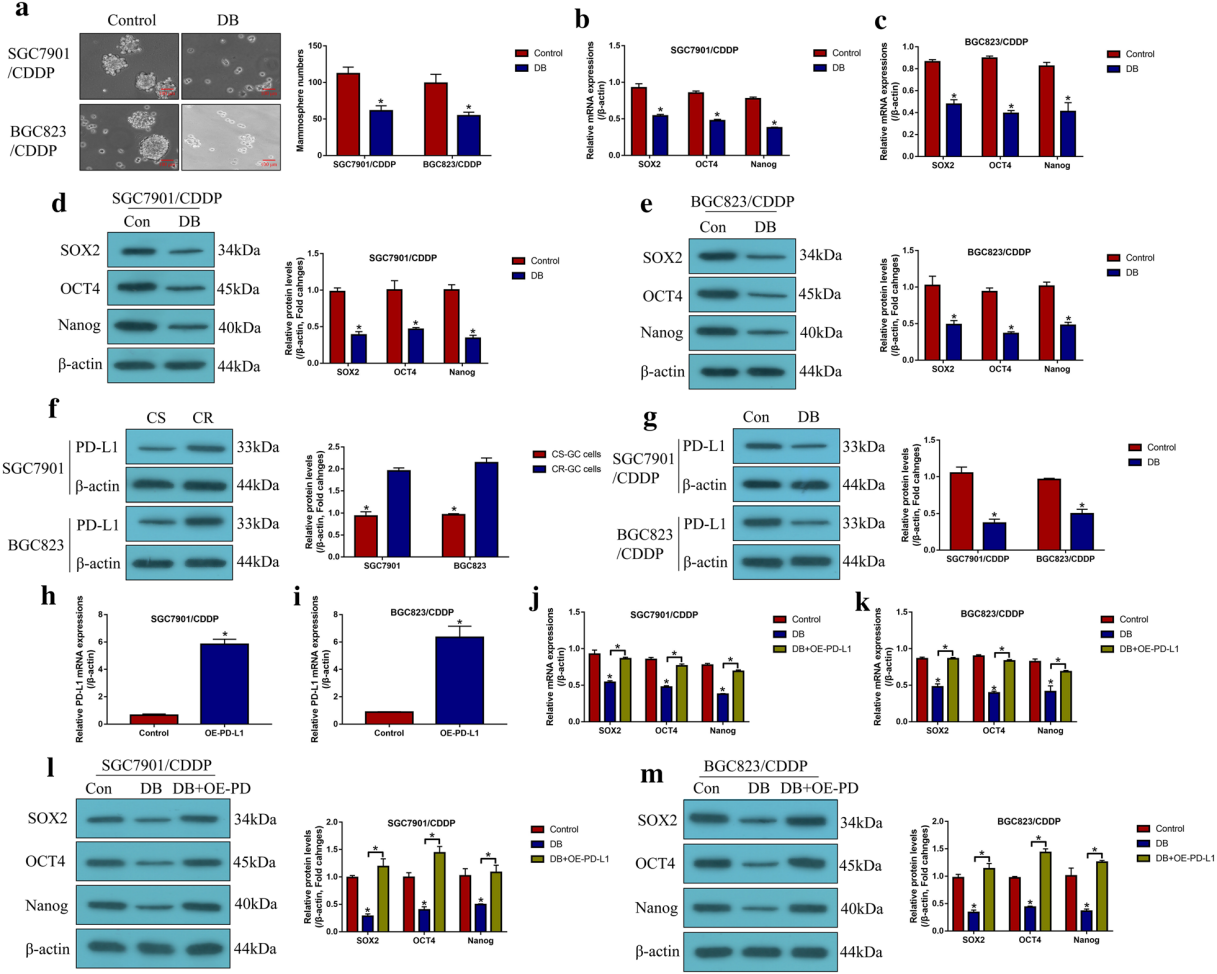
Low-dose DB downregulated PD-L1 to inhibit CSCs properties in CR-GC cells. The CR-GC cells were administered with low-dose DB for 48 h, **a** spheroid formation abilities were evaluated by the spheroid formation assay, **b**, **c** the mRNA levels and **d**, **e** protein levels of SOX2, OCT4 and Nanog were determined by using the Real-Time qPCR and Western Blot analysis, respectively. **f**, **g** PD-L1 protein levels were determined by conducting Western Blot analysis. **h**, **i** The overexpression vectors for PD-L1 were delivered into CR-GC cells. The expression levels of SOX2, OCT4 and Nanog were examined at both **j**, **k** transcriptional and **l**, **m** translated levels. Each experiment repeated at least three times. *P < 0.05

Since we had proved that low-dose DB sensitized CR-GC cells to cisplatin by triggering NLRP3 mediated pyroptosis, and cisplatin pressure induced generation of CSCs contributed to drug resistance of GC cells. Hence, we next investigated whether low-dose DB affected stemness of CR-GC cells. As shown in Fig. 4a, we found that low-dose DB inhibited spheroid formation abilities in CR-GC cells (*P* < 0.05). Also, the expression levels of SOX2, OCT4 and Nanog were decreased by low-dose DB in CR-GC cells (*P* < 0.05, Fig. 4b–e), suggesting that DB inhibited CSCs properties in CR-GC cells. Based on the information that PD-L1 regulated cancer cell stemness, we validated that PD-L1 was upregulated in CR-GC cells, compared to the corresponding CS-GC cells (*P* < 0.05, Fig. 4f). Interestingly, we proved that low-dose DB negatively regulated PD-L1 in CR-GC cells (*P* < 0.05, Fig. 4g), which enlightened us that low-dose DB might regulate cell stemness in CR-GC cells through PD-L1. To validate the above hypothesis, the PD-L1 overexpression vectors were successfully transfected into CR-GC cells (*P* < 0.05, Fig. 4h, i), and the results showed that upregulation of PD-L1 promoted the stemness signatures (SOX2, OCT4 and Nanog) expressions to improve CSCs properties in DB-treated CR-GC cells (*P* < 0.05, Fig. 4j–m). Consistently, the in vivo extreme limiting dilution analysis (ELDA) results suggested that tumors in DB + OE-PD-L1 group formed at an earlier time point and developed at faster rate than the cells in DB alone group (Additional file 1: Table S4). The above results indicated that low-dose DB inhibited CSCs enrichment in CR-GC cells by downregulating PD-L1 in vitro and in vivo.

### Knock-down of PD-L1 triggered pyroptotic cell death in CR-GC cells through activating NLRP3 inflammasome

**Fig. 5:**
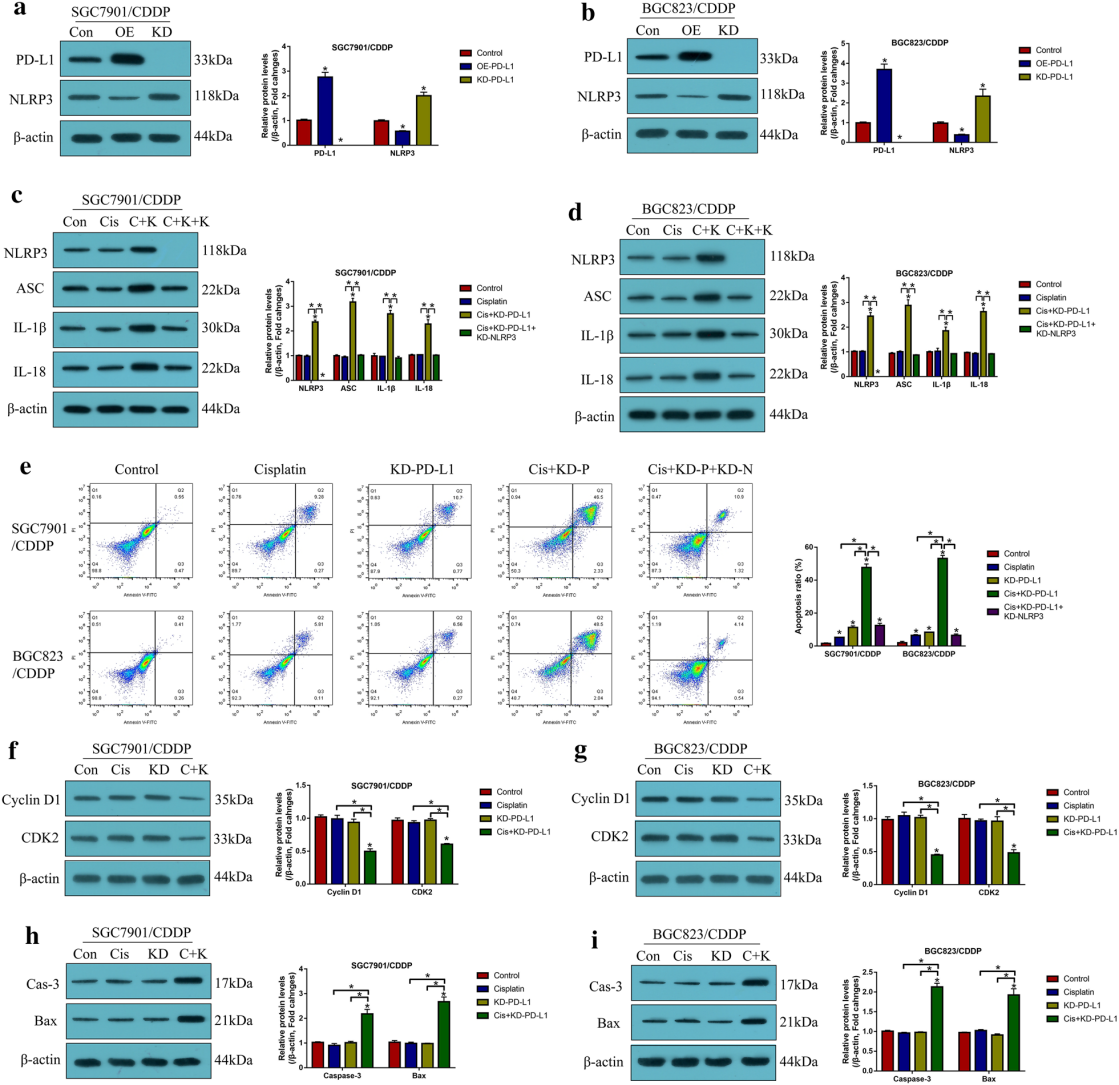
Knock-down of PD-L1 triggered apoptotic and pyroptotic cell death in cisplatin treated CR-GC cells in vitro and in vivo. The expression levels of **a**, **b** PD-L1 and NLRP3, and **c**, **d** pyroptosis associated biomarkers were evaluated by Western Blot analysis. **e** The Annexin V-FITC/PI double staining assay was performed to determine cell apoptosis ratio. Western Blot analysis was conducted to detect the protein levels of **f**, **g** Cyclin D1 and CDK2, and **h**, **i** cleaved Caspase-3 and Bax in mice tumor tissues. (Note: “Con” represented “Control”, “OE” indicated “PD-L1 overexpression”, “KD” represented “PD-L1 silence”, “Cis” indicated “Cisplatin”, “C + K” represented “Cisplatin plus PD-L1 ablation”, “C + K + K” represented “Cisplatin plus PD-L1 ablation plus silencing NLRP3”). Each experiment repeated at least three times. *P < 0.05

Aside from stemness, PD-L1 also directly regulated cell proliferation and death to modulate drug resistance in cancer cells, and recent data suggested that there existed a tumor-intrinsic PD-L1-NLRP3 signaling pathway in tumor cells [38], based on this, as shown in Fig. 5, our further experiments validated that silencing of PD-L1 activated NLRP3 mediated pyroptosis in cisplatin treated CR-GC cells. Mechanistically, the PD-L1 overexpression and downregulation vectors were successfully delivered into CR-GC cells (*P* < 0.05, Fig. 5a, b), and we verified that PD-L1 negatively regulated NLRP3 in CR-GC cells (*P* < 0.05, Fig. 5a, b). In addition, knock-down of PD-L1 increased the expression levels of NLRP3, ASC, IL-1β, IL-18 (*P* < 0.05, Fig. 5c, d), cleaved caspase-1 and N-Gasdermin D (*P* < 0.05, Additional file 1: Figure S4A-D) to facilitate pyroptotic cell death in cisplatin treated CR-GC cells, which were abrogated by silencing NLRP3 (*P* < 0.05, Fig. 5c, d, Additional file 1: Figure S4A-D), suggesting that targeting intrinsic PD-L1/NLRP3 pathway triggered cell pyroptosis in cisplatin-treated CR-GC cells. Also, either cisplatin or PD-L1 ablation alone promoted cell apoptosis in CR-GC cells to a very limit extent (*P* < 0.05, Fig. 5e), which were significantly enhanced by co-treating cells with cisplatin and PD-L1 downregulation (*P* < 0.05, Fig. 5e). Additionally, the promoting effects of cisplatin and silencing PD-L1 co-treatments on cell apoptosis in CR-GC cells were reversed by knocking down NLRP3 (*P* < 0.05, Fig. 5e). Furthermore, the xenograft tumor-bearing mice models were established, and we proved that knock-down of PD-L1 inhibited Cyclin D1 and CDK2 (*P* < 0.05, Fig. 5f, g), and Ki67 (Additional file 1: Figure S2), while promoted Caspase-3 and Bax expressions (*P* < 0.05, Fig. 5h, i) to facilitate cell growth in CR-GC cells in vivo.

### Low-dose DB enhanced the cytotoxic effects of cisplatin on CR-GC cells through downregulating PD-L1

**Fig. 6:**
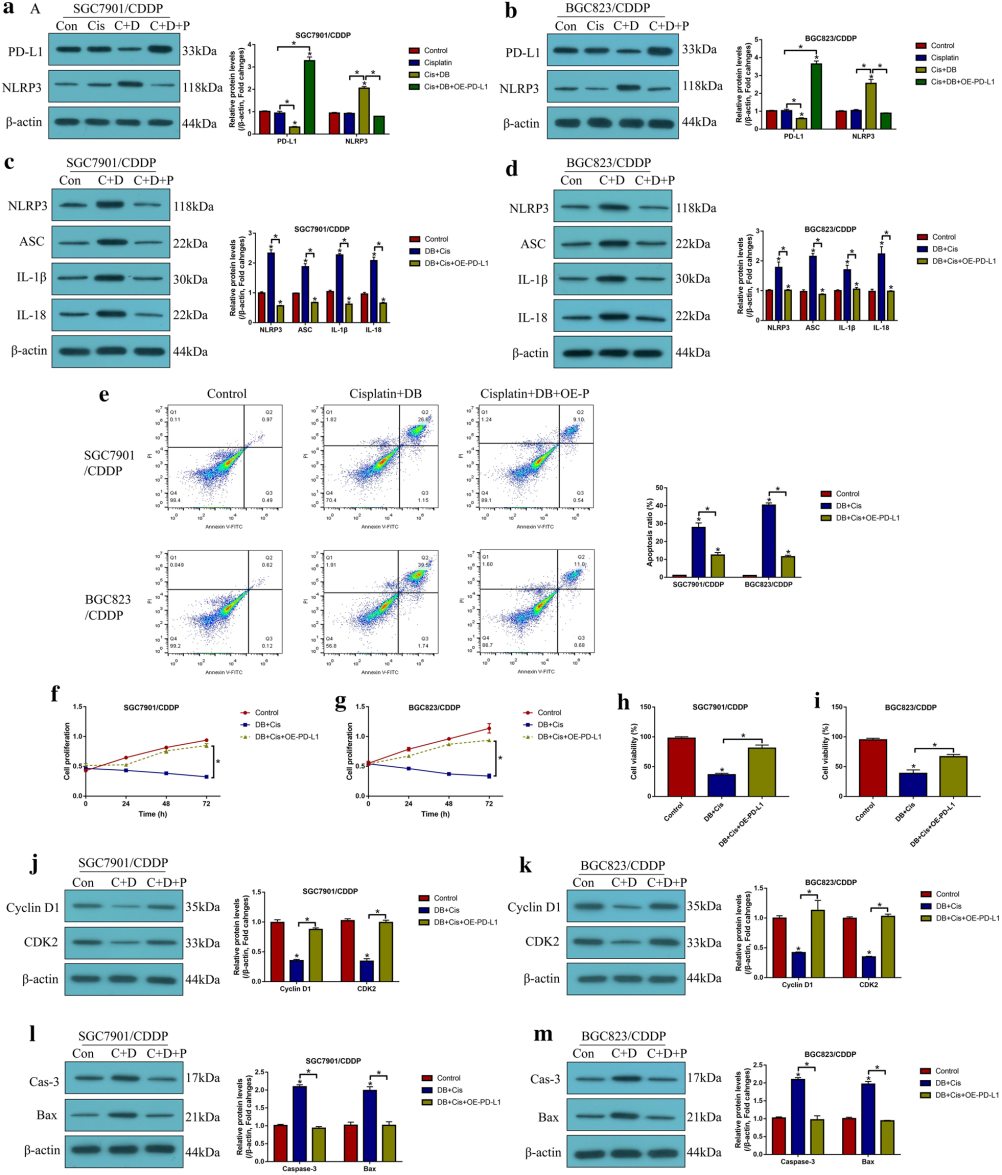
Low-dose DB downregulated PD-L1 to trigger apoptotic and pyroptotic cell death in cisplatin treated CR-GC cells. Western Blot analysis was performed to determine **a**, **b** PD-L1 and NLRP3, and **c**, **d** pyroptosis associated biomarkers. **e** Annexin V-FITC/PI double staining method was used to examine cell apoptosis. **f**, **g** CCK-8 assay was used to measure cell proliferation, and **h**, **i** trypan blue staining assay was employed to determine cell viability. Western Blot analysis was employed to determine the expression levels of **j**, **k** proliferation associated proteins (Cyclin D1 and CDK2) and **l**, **m** apoptosis associated proteins (cleaved Caspase-3 and Bax) in mice tumor tissues. (Note: “Con” indicated “Control”, “Cis” suggested “Cisplatin”, “C + D” indicated “Cisplatin plus DB”, “C + D + P” indicated “Cisplatin plus DB plus PD-L1 overexpression”). Each experiment repeated at least three times. *P < 0.05

Finally, as shown in Fig. 6, we found that low-dose DB sensitized CR-GC cells to cisplatin stimulation through targeting the intrinsic PD-L1/NLRP3 signaling pathway. Functionally, we found that cisplatin alone had little effects on PD-L1 and NLRP3 expressions (*P* > 0.05, Fig. 6a, b), while the promoting effects of cisplatin-DB co-treatment on NLRP3, ASC, IL-1β and IL-18 expressions were abrogated by overexpressing PD-L1 (*P* < 0.05, Fig. 6a–d), implying that DB triggered NLRP3-mediated pyroptotic cell death in cisplatin-treated CR-GC cells by downregulating PD-L1. Furthermore, induction of apoptotic cell death by cisplatin-DB co-treatment was reversed by overexpressing PD-L1 (*P* < 0.05, Fig. 6e), and the inhibiting effects of DB stimulation on cell proliferation (*P* < 0.05, Fig. 6f, g) and viability (*P* < 0.05, Fig. 6h, i) in cisplatin-treated CR-GC cells were also restored by upregulating PD-L1. In addition, the above cellular results were validated by the following in vivo results, and the results showed that overexpression of PD-L1 increased Cyclin D1 and CDK2 expression levels, while inhibited cleaved Caspase-3 and Bax expressions in the tumor tissues collected from xenograft mice administered with DB-Cisplatin combined treatment (*P* < 0.05, Fig. 6j–m). Moreover, we noticed that PD-L1 recombinant protein could not reverse the inhibiting effects of cisplatin-DB co-treatment on cell proliferation (Additional file 1: Figure S6A, B) and viability (Additional file 1: Figure S6C, D).

## Discussion

Continuous cisplatin exposure brought gastric cancer (GC) cells with chemo-resistance to this chemical drug [9, 39], which seriously limited the therapeutic efficacy of cisplatin for GC treatment in clinic. Recently, researchers agreed that development of new anti-tumor drugs might help to solve this problem [1–3]. Based on our previous work [5], the present study proved that low-dose Diosbulbin-B (DB) was advantageous to improve cisplatin-sensitivity in GC cells, which also avoided the side effects that high-dose DB induced hepatotoxicity [8]. Mechanistically, the established cisplatin-resistant GC (CR-GC) cells were obtained, and our results showed that either cisplatin or low-dose DB alone had little effects on cell proliferation and viability in CR-GC cells. In addition, we surprisingly found that DB-cisplatin combined strategy significantly promoted cell apoptosis in CR-GC cells, implying that low-dose DB triggered apoptotic cell death to increase the cytotoxic effects of cisplatin on CR-GC cells, which was partly supported by the previous data that DB was able to induce cell apoptosis [5, 8]. Consistently, by establishing the xenograft mice models, we proved that cisplatin-DB combination therapy inhibited tumorigenesis, induced cell cycle arrest and apoptosis, and inhibited cell growth of CR-GC cells in vivo. The above results suggested that low-dose DB increased cisplatin-sensitivity in CR-GC cells.

Next, we investigated the potential underlying mechanisms, and the results showed that low-dose DB triggered NLRP3 mediated pyroptotic cell death in cisplatin treated CR-GC cells. Specifically, by pre-treating CR-GC cells with the inhibitors for pyroptosis, apoptosis, autophagy and ferroptosis, we surprisingly found that blockage of apoptosis and pyroptosis, instead of other types of cell death, rescued cell proliferation and viability, and inhibited cell apoptosis in cisplatin-DB co-treated CR-GC cells. Also, cisplatin-DB co-treatment increased NLRP3, ASC, IL-1β, IL-18, cleaved caspase-1 and N-Gasdermin D expression levels to promote cell pyroptosis in CR-GC cells, suggesting that low-dose DB triggered both pyroptotic and apoptotic cell death in cisplatin treated CR-GC cells. In addition, previous data suggested that there existed crosstalk between cell pyroptosis and apoptosis [40, 41], and our data showed that the pyroptosis inhibitor (NSA) inhibited cell apoptosis, while the apoptosis inhibitor (Z-VAD-FMK) had little effects on pyroptotic cell death in cisplatin-DB co-treated CR-GC cells, suggesting that low-dose DB induced cell pyroptosis to promote cell death in cisplatin stimulated CR-GC cells. Consistently, knock-down of NLRP3 abrogated the promoting effects of cisplatin-DB combined treatment on cell apoptosis in CR-GC cells, which were in accordance with the previous studies [40, 42, 43]. Furthermore, the intrinsic PD-L1/NLRP3 pathway had been reported in the previous studies [31, 32], and we validated that low-dose DB inhibited PD-L1 expressions to activate NLRP3 mediated cell pyroptosis in cisplatin treated CR-GC cells. Notably, the inhibiting effects of cisplatin-DB co-treatment on cell proliferation and viability in CR-GC cells were reversed by overexpressing PD-L1, instead of the PD-L1 recombinant protein. The possible reason was that, PD-L1 recombinant protein cannot be incorporated into the CR-GC cells, which cannot approach the cytoplasm of the cells to regulate the NLRP3-pyroptosis pathway. In addition, there existed no receptors for PD-L1 to regulate the NLRP3-pyroptosis pathway. The above two reasons made the DB-cisplatin co-treated CR-GC cells irresponsive to PD-L1 recombinant protein treatment. However, this issue still needed to be investigated in our future work.

Cancer stem cells (CSCs) contributed to metastasis, recurrence and drug resistance in cancers [11–14], and previous data suggested that CSCs enrichment contributed to cisplatin-resistance in GC cells [17, 18]. Therefore, elimination of CSCs will help to improve chemo-sensitivity in GC cells [19]. As expected, the present study found that CSCs tended to be enriched in CR-GC cells and acquired cisplatin-resistant GC (ACR-GC) cells, compared to their corresponding parental CS-GC cells, which were in keeping with the previous work [17, 18] and reflected that continuous cisplatin pressure promote CSCs generation in GC cells. Interestingly, we found that low-dose DB inhibited spheroid formation and the expressions of stemness signatures to eliminate CSCs in CR-GC cells, indicating that low-dose DB treatment was a novel strategy to enhance cisplatin-sensitivity in CR-GC cells by inhibiting CSCs properties. Furthermore, PD-L1 regulated CSCs features in colorectal cancer [25], breast cancer [33] and pancreatic cancer [44], and this study validated that low-dose DB negatively regulated PD-L1 to inhibit cell stemness in CR-GC cells. Of note, previous publications suggested that NLRP3 inflammasome pathway was closely associated with CSCs homing, engraftment and trafficking [45, 46], and we will investigate this issue in GC in our future work.

Taken together, this study validated that low-dose DB sensitized CR-GC cells to cisplatin through regulating the tumor intrinsic PD-L1/NLRP3 mediated pyroptosis and CSCs properties, and provided evidences to support that low-dose DB was a novel strategy to treat GC by combining with traditional chemotherapeutic drugs in clinic. However, future clinical experiments are still needed to validate the above pre-clinical results.

## Supplementary Information

**Additional file 1: Figure S1.** Low-dose DB decreased Ki67 expression levels in cisplatin treated mice tumor tissues, examined by immunohistochemistry. **Figure S2.** Knock-down of PD-L1 inhibited Ki67 expressions in cisplatin treated mice tumor tissues, examined by immunohistochemistry. **Figure S3.** The additional representative tumor images for Fig. 1f. **Figure S4.** Western Blot analysis was performed to examine the expression levels of cleaved caspase-3 and N-Gasdermin D (N-GSDMD) in (A, B) SGC7901/CDDP cells and (C, D) BGC823/CDDP cells. *P < 0.05. **Figure S5.** The expression levels of p-MLKL were examined by Western Blot analysis. **Figure S6.** The effects of PD-L1 recombinant protein treatment on (A-B) cell proliferation and (C-D) viability. Individual experiment was repeated at least 3 times, and *P < 0.05. **Table S1.** The sequence information for vectors construction. **Table S2.** Primer sequences for Real-Time qPCR. **Table S3.** The detailed information of antibodies for Western Blot analysis. **Table S4.** In vivo tumor formation assay by the extreme limiting dilution analysis (ELDA).

## Data Availability

The datasets supporting the conclusions of this article had been included within the manuscript.
