# Supplementary material for: Low-dose Diosbulbin-B (DB) activates tumor-intrinsic PD-L1/NLRP3 signaling pathway mediated pyroptotic cell death to increase cisplatin-sensitivity in gastric cancer (GC)
Source: Cell Biosci. 2021 Feb 12;11:38. doi: 10.1186/s13578-021-00548-x (PMC7881658; doi:10.1186/s13578-021-00548-x)
Supplement: Supplementary file 1 — Additional file 1: Figure S1. Low-dose DB decreased Ki67 expression levels in cisplatin treated mice tumor tissues, examined by immunohistochemistry. Figure S2. Knock-down of PD-L1 inhibited Ki67 expressions in cisplatin treated mice tumor tissues, examined by immunohistochemistry. Figure S3. The additional representative tumor images for Fig. 1f. Figure S4. Western Blot analysis was performed to examine the expression levels of cleaved caspase-3 and N-Gasdermin D (N-GSDMD) in (A, B) SGC7901/CDDP cells and (C, D) BGC823/CDDP cells. *P < 0.05. Figure S5. The expression levels of p-MLKL were examined by Western Blot analysis. Figure S6. The effects of PD-L1 recombinant protein treatment on (A-B) cell proliferation and (C-D) viability. Individual experiment was repeated at least 3 times, and *P < 0.05. Table S1. The sequence information for vectors construction. Table S2. Primer sequences for Real-Time qPCR. Table S3. The detailed information of antibodies for Western Blot analysis. Table S4. In vivo tumor formation assay by the extreme limiting dilution analysis (ELDA). [file 13578_2021_548_MOESM1_ESM.docx]

**Additional Figures and Figure legends**


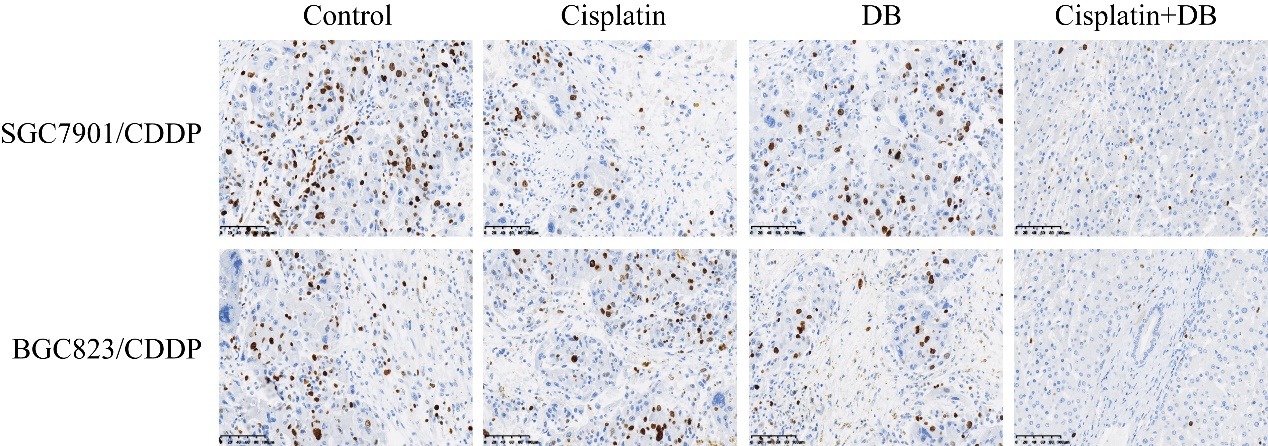


**Figure S1.** Low-dose DB decreased Ki67 expression levels in cisplatin treated mice tumor tissues, examined by immunohistochemistry.


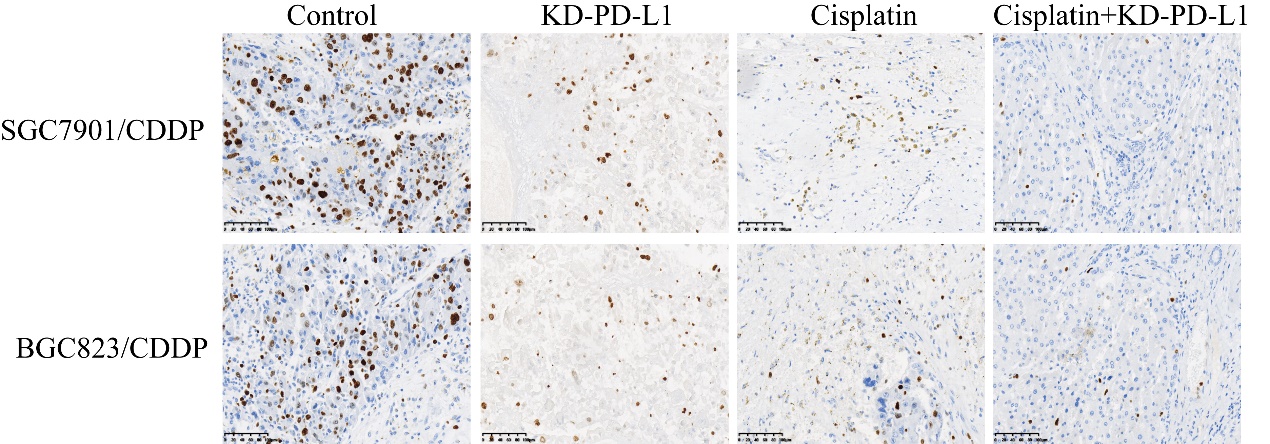


**Figure S2.** Knock-down of PD-L1 inhibited Ki67 expressions in cisplatin treated mice tumor tissues, examined by immunohistochemistry.


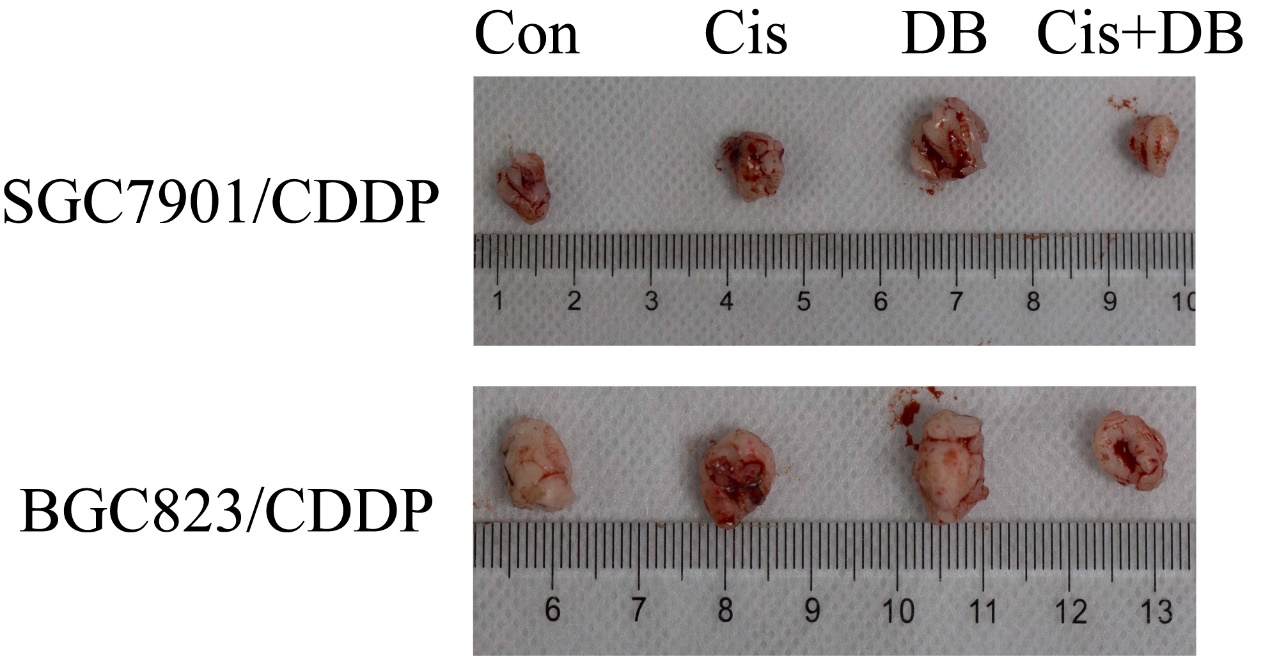


**Figure S3.** The additional representative tumor images for Figure 1F.


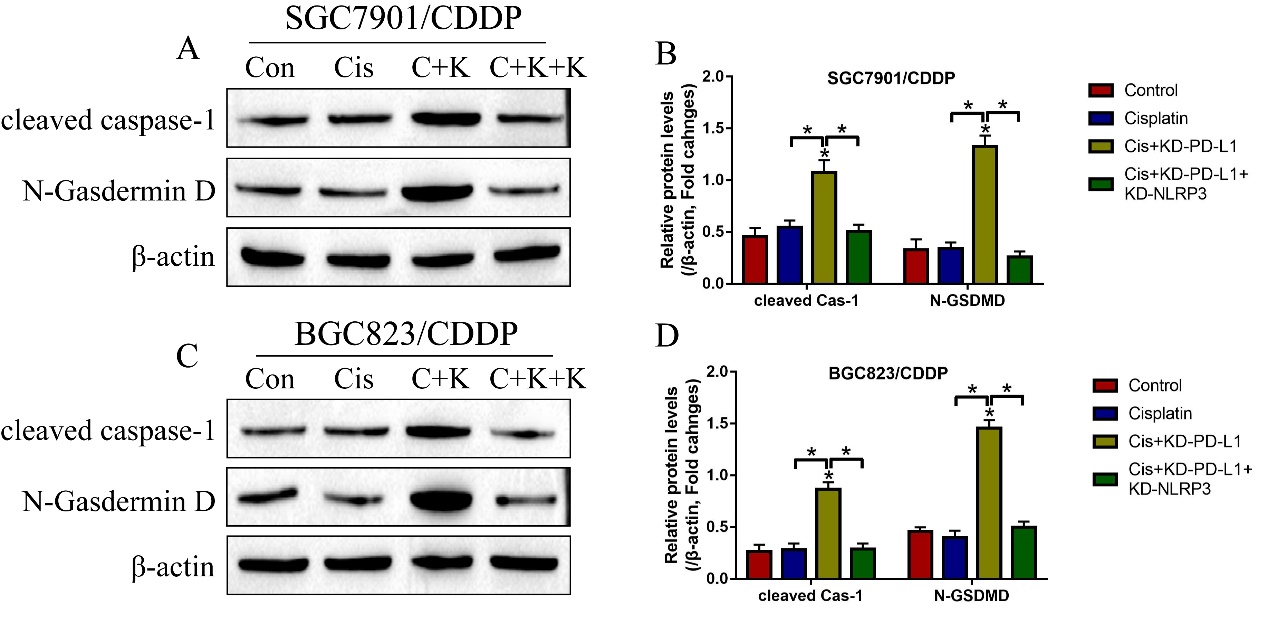


**Figure S4.** Western Blot analysis was performed to examine the expression levels of cleaved caspase-3 and N-Gasdermin D (N-GSDMD) in (A, B) SGC7901/CDDP cells and (C, D) BGC823/CDDP cells. **P* < 0.05.


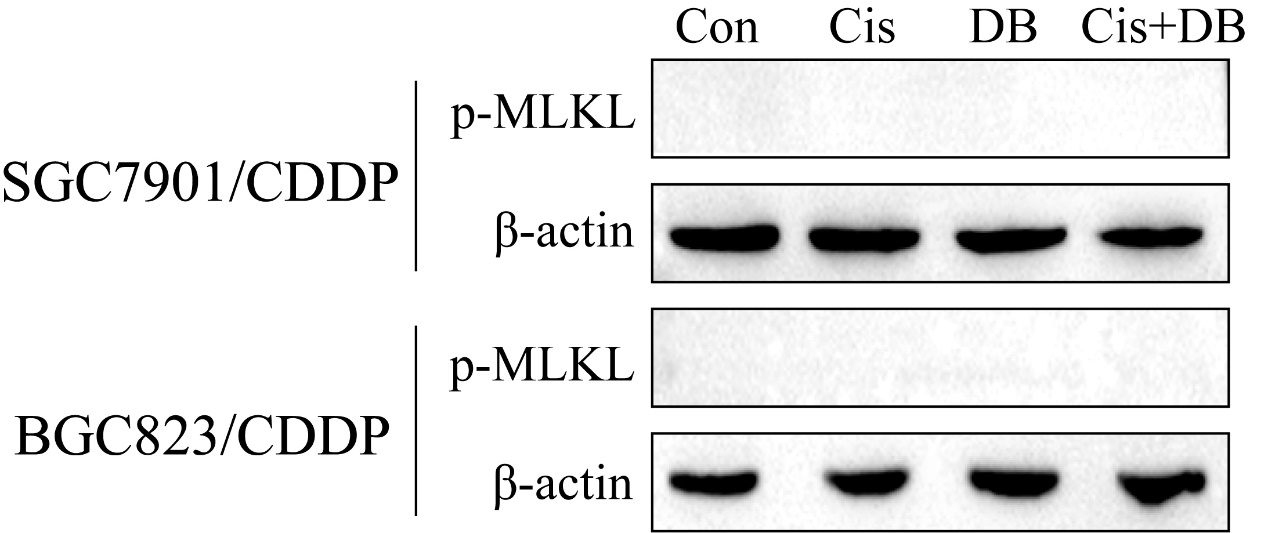


**Figure S5.** The expression levels of p-MLKL were examined by Western Blot analysis.


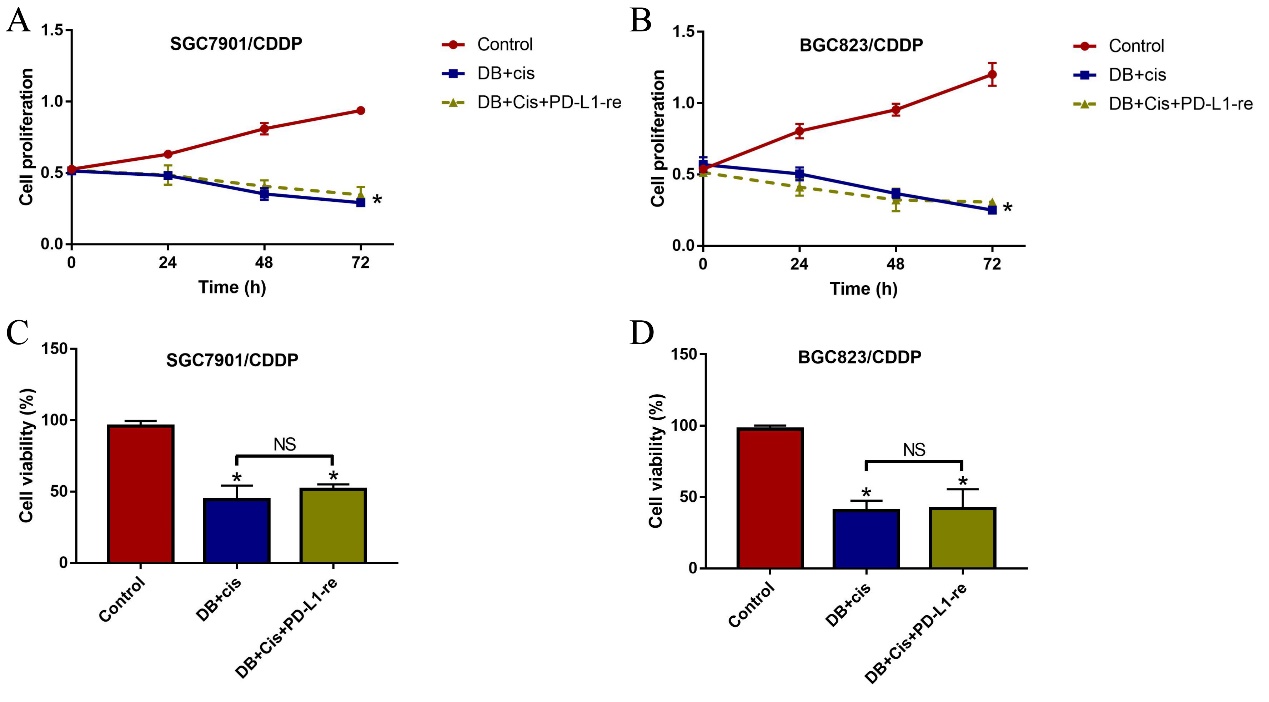


**Figure S6.** The effects of PD-L1 recombinant protein treatment on (A-B) cell proliferation and (C-D) viability. Individual experiment was repeated at least 3 times, and **P* < 0.05.

**Additional Tables and table legends**

**Table S1. The sequence information for vectors construction.**

|  | **Sequence information** |
| --- | --- |
| Si-PD-L1 | Forward: 5’-GGAUAAGAACAUUAUUCAAdTdT-3’  Reverse: 5’-UUGAAUAAUGUUCUUAUCCdTdT-3’ |
| Si-NLRP3 | Forward: 5’- GCAGGAACAACUUGCAGAUGAGUCTTGCUCCU-3’  Reverse: 5’-AGGAGACUCAGCGAAGAAACAAGUUCCUGC-3’ |
| OE-PD-L1 | Forward: 5’-CCGGAATTCGGCATTCCAGAAAGATGAGGAT  ATT-3’  Reverse: 5’-AAGGAAAAAAGCGGCCGCTTGTCACGCTCA  GCCCCGAT-3’ |

**Table S2. Primer sequences for Real-Time qPCR.**

| Gene | Primer sequences (strand) |
| --- | --- |
| β-actin | Forward: 5’-CTCCATCCTGGCCTCGCTGT-3’  Reverse: 5’-GCTGCTACCTTCACCGTTCC-3’ |
| NLRP3 | Forward: 5’- GGACTATTTCCCCAAGATTG-3’  Reverse: 5’- ACTCCACCCGATGACAGTT-3’ |
| OCT4 | Forward: 5’-AGCGATCAAGCAGCGACTA-3’  Reverse: 5’-GGAAAGGGACCGAGGAGTA-3’ |
| SOX2 | Forward: 5’-CATCACCCACAGCAAATGAC-3’  Reverse: 5’-CAAAGCTCCTACCGTACCACT-3’ |
| Nanog | Forward: 5’-GCAGGCAACTCACTTTATCC -3’  Reverse: 5’-CCCACAAATCACAGGCATAG-3’ |
| PD-L1 | Forward: 5’-TGGCATTTGCTGAACGCATTT-3’  Reverse: 5’-TGCAGCCAGGTCTAATTGTTTT -3’ |

**Table S3. The detailed information of antibodies for Western Blot analysis.**

| Antibodies | Catalog No. | Working concentrations | Company |
| --- | --- | --- | --- |
| PD-L1 | #ab213524 | 1:1500 | Abcam, UK |
| SOX2 | #ab97959 | 1:2000 | Abcam, UK |
| OCT4 | #ab200834 | 1:1500 | Abcam, UK |
| β-actin | #ab8226 | 1:1500 | Abcam, UK |
| Cleaved Caspase-1 | #4199 | 1:800 | Cell Signaling Technology |
| Gasdermin D-N | #96458 | 1:1000 | Cell Signaling Technology |
| Nanog | #ab241542 | 1:2000 | Abcam, UK |

**Table S4. *In vivo* tumor formation assay by the extreme limiting dilution analysis (ELDA).**

|  |  | **Mice number of tumors/Mice number of injections** | | | | | | | | | |
| --- | --- | --- | --- | --- | --- | --- | --- | --- | --- | --- | --- |
|  |  | **Days post-injection** | | | | | | | | | |
| **Cell No.** | **Groups** | **8** | **11** | **14** | **17** | **20** | **23** | **26** | **29** | **32** | **35** |
| **1 × 10^6^** | **Con** | 4/8 | 6/8 | 7/8 | 8/8 | 8/8 | 8/8 | **-** | **-** | **-** | **-** |
|  | **DB** | 1/8 | 3/8 | 4/8 | 6/8 | 7/8 | 8/8 | **-** | **-** | **-** | **-** |
|  | **DB+PD** | 3/8 | 5/8 | 8/8 | 8/8 | 8/8 | 8/8 | **-** | **-** | **-** | **-** |
| **1 × 10^5^** | **Con** | **-** | 3/8 | 4/8 | 4/8 | 5/8 | 6/8 | 8/8 | 8/8 | 8/8 | 8/8 |
|  | **DB** | **-** | **-** | **-** | 1/8 | 3/8 | 4/8 | 4/8 | 5/8 | 5/8 | 6/8 |
|  | **DB+PD** | **-** | **-** | 2/8 | 4/8 | 4/8 | 6/8 | 6/8 | 7/8 | 7/8 | 7/8 |
| **1 × 10^4^** | **Con** | **-** | **-** | **-** | **-** | 3/8 | 4/8 | 4/8 | 6/8 | 7/8 | 7/8 |
|  | **DB** | **-** | **-** | **-** | 1/8 | 1/8 | 1/8 | 3/8 | 3/8 | 4/8 | 4/8 |
|  | **DB+PD** | **-** | **-** | **-** | **-** | **-** | 3/8 | 5/8 | 6/8 | 6/8 | 8/8 |
